# Supplementary material for: Multilayer Perceptron-Based Real-Time Intradialytic Hypotension Prediction Using Patient Baseline Information and Heart-Rate Variation
Source: Int J Environ Res Public Health. 2022 Aug 20;19(16):10373. doi: 10.3390/ijerph191610373 (PMC9408052; doi:10.3390/ijerph191610373)
Supplement: Supplementary file 1 [file ijerph-19-10373-s001.zip › ijerph-1830375-supplementary.pdf]

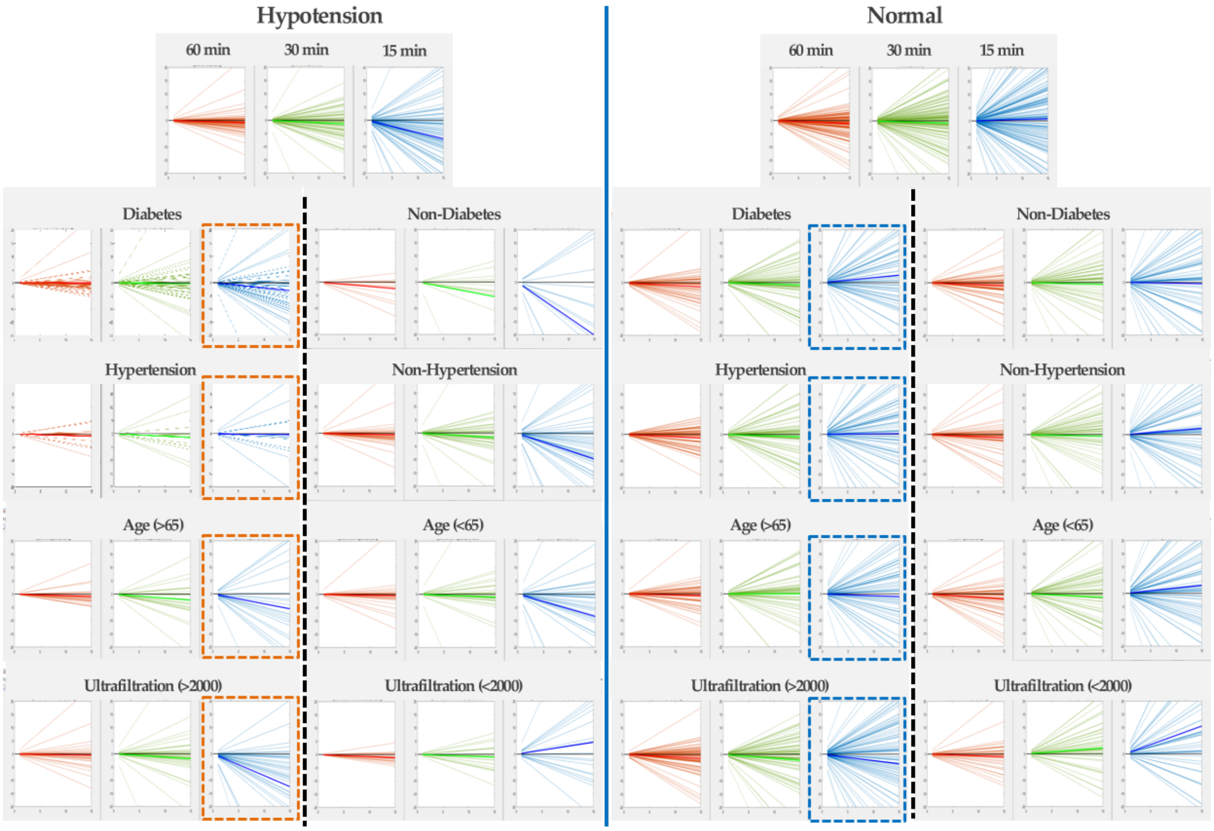

Figure S1. Changes in HR slope for IDH and normal (non-IDH) patients according to 60 min data by patient baseline information before the onset of IDH.

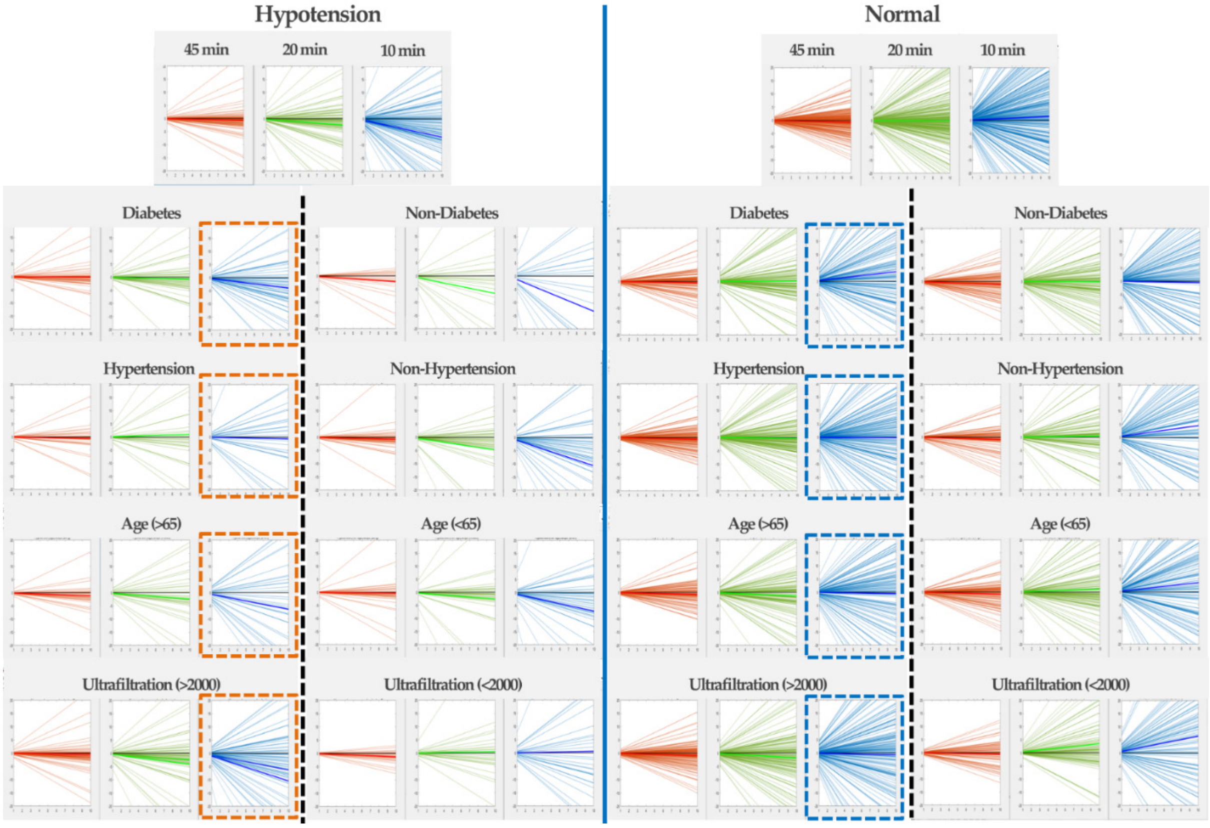

Figure S2. Changes in HR slope for IDH and normal (non-IDH) patients according to 45 min data by patient baseline information before the onset of IDH.

Table S1. HR slope values for IDH and normal (non-IDH) patients according to patient baseline information 60 min before IDH onset.

	Hypotension						Normal					
	60min	30min	15min	60min	30min	15min	60min	30min	15min	60min	30min	15min
Underlying disease [1]	Diabetes			Non-diabetes			Diabetes			Non-diabetes		
Mean slope	-0.00872	-0.02342	-0.2023	-0.16395	-0.36813	-1.3420	-0.10618	-0.05459	0.18847	-0.07546	-0.04555	-0.03470
Num. of positive slopes	13	16	12	3	6	4	28	39	45	36	56	53
Num. of negative slopes	36	33	37	13	10	12	55	44	38	47	27	30
% of negative slopes	73.47	67.35	75.51	81.25	62.50	75.00	66.27	53.01	45.78	56.63	32.53	36.14
Underlying disease	Hypertension			Non-hypertension			Hypertension			Non-hypertension		
Mean slope	-0.05603	-0.09166	-0.02140	-0.04329	-0.11563	-0.6335	-0.09309	-0.05636	0.01554	-0.08785	-0.04186	0.15694
Num. of positive slopes	5	3	4	11	20	12	39	48	53	25	47	45
Num. of negative slopes	11	13	12	38	29	37	55	46	41	47	25	27
% of negative slopes	68.75	81.25	75.00	77.55	59.18	75.51	58.51	48.94	43.62	65.28	34.72	37.50
Underlying disease	Age			Non-age			Age			Non-age		
Mean slope	-0.06055	-0.15182	-0.37490	-0.03640	-0.07982	-0.55950	-0.04493	-0.00005	-0.06980	-0.13052	-0.09335	0.20373
Num. of positive slopes	6	9	7	10	14	9	36	51	43	28	44	55
Num. of negative slopes	21	18	20	28	24	29	41	26	34	61	45	34
% of negative slopes	77.78	66.67	74.07	73.68	63.16	76.32	53.25	33.77	44.16	68.54	50.56	38.20
Underlying disease	Ultrafiltration			Non-ultrafiltration			Ultrafiltration			Non-ultrafiltration		
Mean slope	-0.03183	-0.12635	-0.83020	-0.07927	-0.07232	0.29893	-0.10334	-0.15194	-0.24260	-0.06623	0.15002	0.70434
Num. of positive slopes	11	15	6	5	8	10	41	56	55	23	39	43
Num. of negative slopes	34	30	39	15	12	10	69	54	55	33	17	13
% of negative slopes	75.56	66.67	86.67	75.00	60.00	50.00	62.73	49.09	50.00	58.93	30.36	23.21

Table S2. HR slope values for IDH and normal (non-IDH) patients according to patient baseline information 45 min before IDH onset

	Hypotension						Normal					
	45min	20min	10min	45min	20min	10min	45min	20min	10min	45min	20min	10min
Underlying disease [1]	Diabetes			Non-diabetes			Diabetes			Non-diabetes		
Mean slope	-0.00688	-0.07913	-0.395	-0.22461	-0.66304	-1.333	-0.04726	0.0050	0.3648	-0.11348	-0.01303	-0.053
Num. of positive slopes	20	20	15	8	8	11	81	82	105	63	88	99
Num. of negative slopes	39	39	44	17	17	14	102	101	78	108	83	72
% of negative slopes	66.10	66.10	74.58	68	68	56	55.74	55.19	42.62	63.16	48.54	42.11
Underlying disease	Hypertension			Non-hypertension			Hypertension			Non-hypertension		
Mean slope	-0.05687	0.094167	-0.076	-0.08126	-0.47749	-0.061	-0.06936	-0.03107	-0.019	-0.09473	0.039103	0.448
Num. of positive slopes	13	11	15	15	17	11	84	89	114	60	81	90
Num. of negative slopes	20	22	18	36	34	40	132	127	102	78	57	48
% of negative slopes	60.61	66.67	54.55	70.59	66.67	78.43	61.11	58.80	47.22	56.52	41.30	34.78
Underlying disease	Age			Non-age			Age			Non-age		
Mean slope	-0.1150	-0.2413	-0.628	-0.03588	-0.26254	-0.712	-0.0714	-0.13159	-0.0527	-0.08626	0.11049	0.356
Num. of positive slopes	13	12	14	15	16	12	66	86	90	78	84	114
Num. of negative slopes	25	26	24	31	30	34	101	81	77	109	103	73
% of negative slopes	65.79	68.42	63.16	67.39	65.22	73.91	60.48	48.50	46.11	58.29	55.08	39.04
Underlying disease	Ultrafiltration			Non-ultrafiltration			Ultrafiltration			Non-ultrafiltration		
Mean slope	-0.04305	-0.41123	-1.044	-0.12895	0.063722	0.0654	-0.08773	-0.17807	-0.074	-0.06229	0.344998	0.6382
Num. of positive slopes	18	15	14	10	13	12	88	101	130	56	69	74
Num. of negative slopes	38	41	42	18	15	16	148	135	106	62	49	44
% of negative slopes	67.86	73.21	75	64.29	53.57	57.14	62.71	57.20	44.92	52.54	41.53	37.29
